# Epithelial Wnt secretion drives the progression of inflammation-induced colon carcinoma in murine model

**DOI:** 10.1016/j.isci.2021.103369

**Published:** 2021-10-28

**Authors:** Bahar Degirmenci, Cansu Dincer, Habibe Cansu Demirel, Linda Berkova, Andreas E. Moor, Abdullah Kahraman, George Hausmann, Michel Aguet, Nurcan Tuncbag, Tomas Valenta, Konrad Basler

**Affiliations:** 1 Department of Molecular Life Sciences, University of Zurich, Winterthurerstrasse. 190, CH-8057 Zurich, Switzerland; 2 Department of Molecular Biology and Genetics, Bilkent University, Ankara, Turkey; 3 National Nanotechnology Center, Bilkent University, Ankara, Turkey; 4 Graduate School of Informatics, Department of Health Informatics, METU, Ankara, Turkey; 5 Institute of Molecular Genetics of the ASCR, v. v. i., Vídeňská 1083142 20, Prague 4, Czech Republic; 6 Swiss Institute for Experimental Cancer Research (ISREC), Ecole Polytechnique Fédérale de Lausanne (EPFL), School of Life Sciences, 1015 Lausanne, Switzerland; 7 Department of Biosystems Science and Engineering, ETH Zürich, Basel, Switzerland; 8 Department of Pathology and Molecular Pathology, University Hospital Zurich, Zurich, Switzerland

**Keywords:** Molecular physiology, Immunology, Cell biology, Cancer, Omics

## Abstract

Colon cancer is initiated by stem cells that escape the strict control. This process is often driven through aberrant activation of Wnt signaling by mutations in components acting downstream of the receptor complex that unfetter tumor cells from the need for Wnts. Here we describe a class of colon cancer that does not depend on mutated core components of the Wnt pathway. Genetically blocking Wnt secretion from epithelial cells of such tumors results in apoptosis, reduced expression of colon cancer markers, followed by enhanced tumor differentiation. In contrast to the normal colonic epithelium, such tumor cells autosecrete Wnts to maintain their uncontrolled proliferative behavior. In humans, we determined certain cases of colon cancers in which the Wnt pathway is hyperactive, but not through mutations in its core components. Our findings illuminate the path in therapy to find further subtypes of Wnt-dependent colon cancer that might be responsive to Wnt secretion inhibitors.

## Highlights

• Acquired expression of epithelial Wnts can drive colon cancer in murine AOM/DSS model
• Blocking epithelial Wnt-secretion induces apoptosis of AOM/DSS cancer cells
• The loss of epithelial Wnts promotes differentiation of Wnt-dependent colon tumors
• Organoids derived from AOM/DSS cancer depend on self-autonomously secreted Wnts

## Graphical abstract

**Figure:**
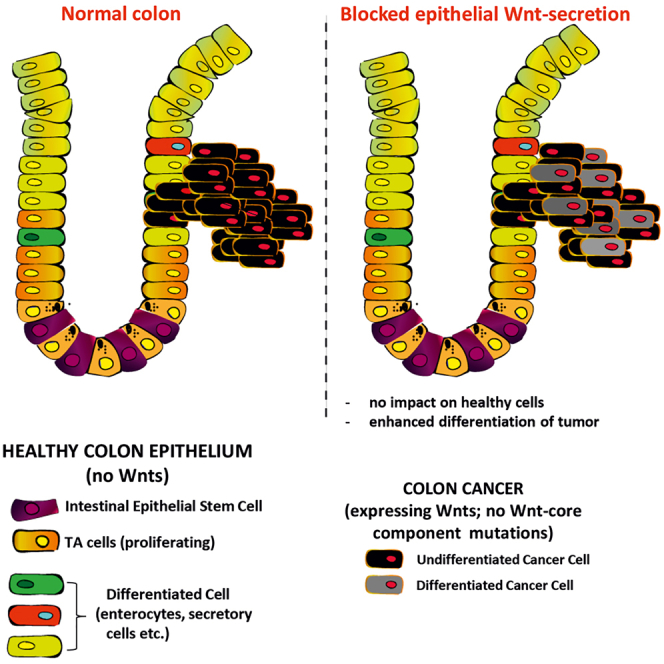

## Introduction

The human colon epithelium harbors stem cells that are responsible for its homeostasis. Tumorigenesis arises when such intestinal epithelial stem (IES) cells escape the tight control by their niche. According to the accepted notion, a single cancer-initiating cell proliferates to form a clonal population by a stepwise process during which distinct mutations are acquired that lead to cancer (Curtius et al., 2018). Various studies in colorectal cancer (CRC) have demonstrated that mutations in the Wnt/β-catenin signaling pathway are the key driving force for the initiation and progression of CRC. More than 90% of the cases of sporadic colonic tumors harbor mutations in the core pathway components APC and/or β-catenin (CTNNB1) (Network CGA, 2012; Sanchez-Vega et al., 2018). The predominance of mutations downstream of the Wnt receptor complexes has been a major hurdle to developing therapeutics for CRC. Indeed, Porcupine (Porcn) inhibitors that have emerged as promising therapy in other cancers failed to demonstrate efficacy in CRCs, mostly due to the downstream position of the causal mutations (Liu et al., 2013). Porcn is an acyltransferase that palmitoylates the Wnt ligands and is required for their secretion (Zhai et al., 2004). Recently, a new class of CRCs responding to Porcn inhibitors was characterized; however, the response was confined to cases with mutated RSPO3 and RNF43/ZNRF3 genes (Koo et al., 2015; Madan et al., 2016). These findings serve as precedence and rationale to identify further subtypes of Wnt-dependent CRCs that might be responsive to such inhibitors.

## Results

To explore this notion, we utilized gene profiling to cluster tumors of patients according to concordant mutations in the Wnt/β-catenin signaling pathway and to define the expression of Wnt ligands. The analysis uncovered a surprising signature of tumors that express Wnt ligands and lack mutations in APC, β-catenin, and other transduction components. A similar signature was detected in a mouse model of chemically induced colon cancer (AOM-DSS): whereas no Wnt ligands are expressed in the healthy colon epithelium, such cancer cells expressed several Wnt genes but did not contain detectable mutations in Wnt/β-catenin pathway components. Using conditional abrogation of Wntless, a multitransmembrane protein required for Wnt secretion, a critical role could be ascribed to these Wnts.

To assess the potential role of Wnt ligands in CRC, we analyzed the data from patients who do not harbor mutations in one of following core components of the Wnt pathway: APC, CTNNB1, RNF43, ZNRF3, TCF7L2 (TCF4), FZD1, FZD3, DKK3, and LRP6. In this subset of patients the expression of the various Wnt ligands was robust (Figures S1A and S1B); some of them are not expressed by healthy colon, for example, Wnt7a and Wnt10a (Farin et al., 2012; Holcombe et al., 2002). Elevated Wnt expression is also observed in patients having mutations in core Wnt pathway components (Figure S1C). The role of acquired Wnt secretion in such type of colon cancer cells was already indicated (Voloshanenko et al., 2013). In contrast, the function of Wnts in colon tumors having intact core Wnt components remains unknown. These tumors may represent a broader spectrum with various contribution of inflammation to their induction and progression. In a further step we focused to probe the role of acquired Wnt secretion using well-defined and established murine model of inflammation-induced colon cancer (azoxymethane/dextran sulfate sodium [AOM/DSS]) having analogy also in human patients. As reported, despite roughly 5% of human CRCs being associated to chronic inflammation, AOM/DSS model is also relevant to many aspects to sporadic CRCs (Schmitt and Greten, 2021).

**Figure 1:**
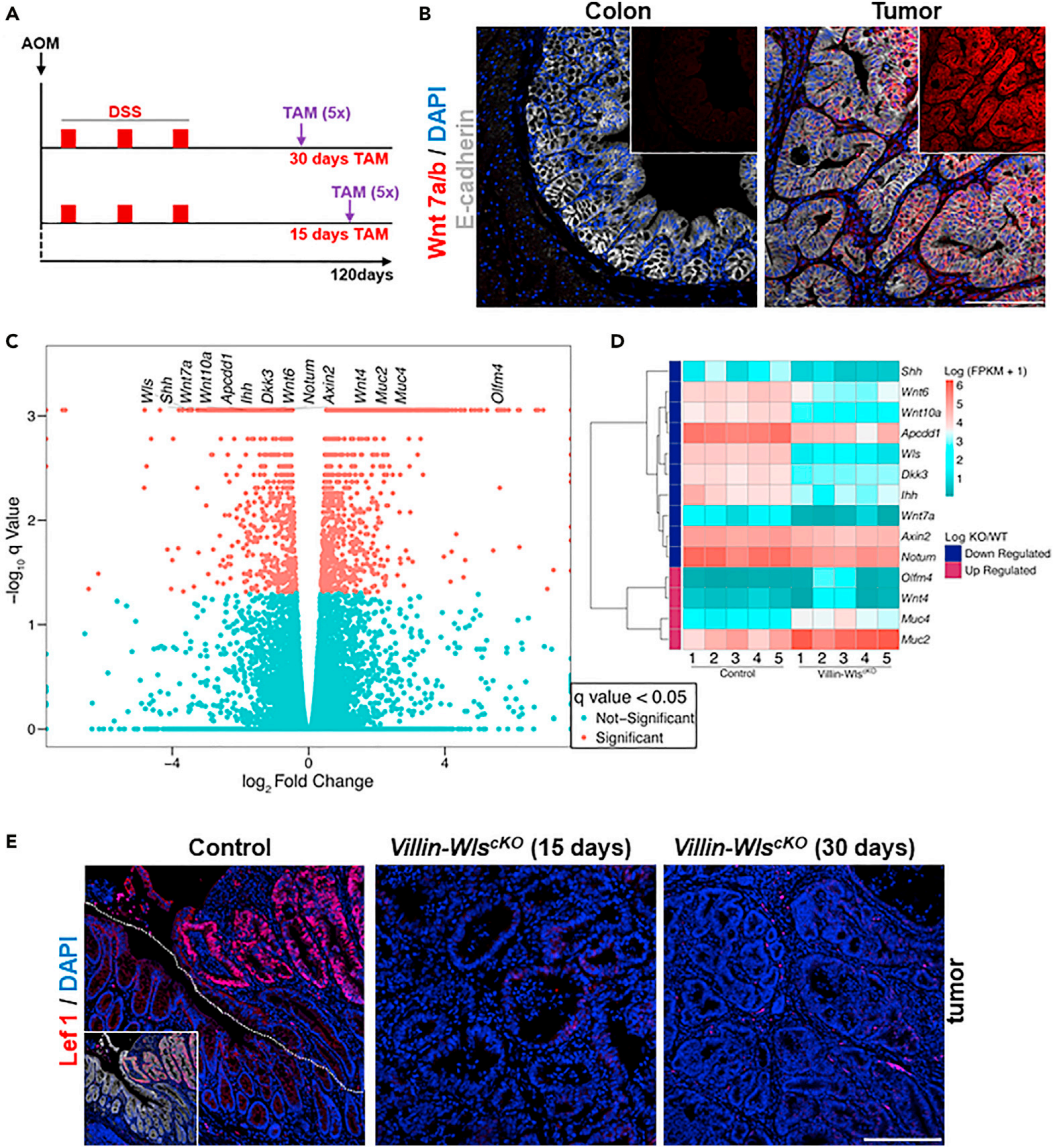
Progression of AOM/DSS colon cancer depends on Wnt ligands secreted from tumor epithelium (A) Scheme of AOM/DSS administration regimen with two strategies of tamoxifen application. Tamoxifen was injected five times in mice in which the tumors were already established. (B) In contrast to healthy colon epithelium, AOM/DSS tumor epithelium express Wnt7a/b. Immunohistochemistry for Wnt7a/b (red); E-cadherin (gray) marks epithelial cells, and DAPI (blue) denotes nuclei. Insets show Wnt7a/b staining (red). (C) Blocking epithelial Wnt secretion in already established AOM/DSS tumors reduced expression of Wnt targets (Apcdd1, Notum, Axin2); markers of differentiated cells (Muc2, Muc4) and negative regulators of colon cancer (Olfm4) were enhanced. Volcano plot showing summarized comparison of Villin-WlscKO (n = 5 tumors) versus control (n = 5 tumors) AOM/DSS tumors (expressing Wls). To block epithelial Wnt secretion, tamoxifen was injected 30 days before tumor collection. (D) Heatmap showing changes in the expression of the indicated genes 30 days after blocking epithelial Wnt secretion in already established AOM/DSS tumors. Five individual control and 5 Villin-WlscKO tumors are shown. Many regulators of Wnt pathway are simultaneously direct Wnt targets (Apcdd1, Dkk3, Axin2, Notum). Increased expressions of Muc2, Muc4, and Olfm4 suggest more differentiated nature of Villin-WlscKO tumors. Reduction of Shh and Ihh may accompany enhanced differentiation. (E) Expression of colon cancer marker and Wnt target Lef1 is strongly reduced after preventing Wnt secretion from tumor epithelium. Immunohistochemistry: Lef1 (red), DAPI (blue) indicates nuclei. Tumor in control (leftmost panel) is marked by dashed line. Inset in control shows overlap of Lef1 (red) with E-cadherin (gray) marking epithelial cells. No Lef1 is expressed by non-tumor colonic crypts. Time after first tamoxifen injection is indicated. Unless otherwise indicated, the tumors with intact Wnt secretion (no tamoxifen) serve as a control. Scale bars, 100 μm.

AOM and DSS-based treatment is widely used to model CRC in laboratory animals. Using a standard regime, we induced CRC in C57BL/6 mice (Figure 1A). To define the mutational landscape in the tumors, we performed exome sequencing in such tumors and in epithelial organoids derived from these tumors. In contrast to previous reports (Greten et al., 2004; Kohno et al., 2005; Tanaka et al., 2005), we could not detect any mutations in coding regions of core Wnt/β-catenin signaling pathway components, such as APC, Ctnnb1, Znfr3, Rnf43, and others (Figures S2, S3, and Data S1). It is possible that the different backgrounds of mice used account for the difference we observed (C57Bl6 versus FVB or CD-1). A similar background dependence of C57BL/6 animals for AOM-DSS-induced tumors was recently also reported by (Pan et al., 2017).

To understand how tumor formation might have occurred in our animals, we analyzed the transcriptomes of the tumors by RNA sequencing. We found substantial expression of *Wnt4, Wnt6, Wnt7a,* and *Wnt10a* genes in the tumors (Figures 1C and 1D, Data S2). Those Wnts are not expressed by healthy colon epithelium. Moreover, Wnt6, Wnt7a, and Wnt10a are absent in the colon completely (Farin et al., 2012). Particularly intriguing were the levels and expression patterns of Wnt7a/b (Figures 1B and S4), which is clearly expressed by the tumor epithelium and organoids, but not in the healthy epithelium (Figures 1B and S4). Expression of *Wnt7a/b* is maintained in organoids derived from AOM/DSS tumors (Figure S4); such organoids no longer depend on medium containing exogenously added Wnts, whereas healthy colonic organoids depend (Figure 3A).

To explore the functional relevance of Wnt expression in the tumors, we used the Cre/*loxP* system to delete *Wntless (Wls)*, which is essential for the proper secretion of Wnts (Bänziger et al., 2006). We first blocked Wnt secretion in the intestinal epithelium using the inducible *Villin-Cre*^*ERT2*^ driver combined with the conditional *Wls* allele (*Wls*^*flox*^). Preventing Wnt secretion from the intestinal epithelium does not affect intestinal homeostasis or survival of these animals (Degirmenci et al., 2018a, 2018b; Valenta et al., 2016). However, first elimination of Wnt secretion from epithelium followed later by AOM/DSS administration in mice led to loss of vital condition. These animals had to be sacrificed. Presumably therefore, epithelial Wnt ligands are important for the recovery from AOM/DSS administrations.

However, we could use *Villin-Cre*^*ERT2*^ to induce recombination of *Wls*^*flox*^ in already established AOM/DSS tumors (Figure 1A), because the *Villin-Cre*^*ERT2*^ driver is active also in tumor cells. We applied tamoxifen to induce Wls-loss and analyzed the tumors at two distinct time points, 15 days and 30 days, before the tumor collection. Consistent with a loss of Wnt signaling, abrogation of Wnt secretion notably diminished the expression of Lef1, a key CRC marker (Porfiri et al., 1997; Hrckulak et al., 2016; Han et al., 2017) (Figures 1E and S5). Control tumors (expressing Wls) showed strong Lef1 expression in the tumorigenic area and no expression in the healthy part containing normal colon crypts. At both time points Lef1 expression was reduced or absent in *Villin-Wls*^*cKO*^ tumors (Figure 1E). The expression levels of other Wnt target genes *Axin2*, *Notum*, *Apcdd1,* and *Dkk3* (Moor et al., 2015) were also reduced (Figures 1C and 1D). Surprisingly, the levels of *Shh*, a putative positive regulator of Wnt signaling and the factor essential for the survival of cancer stem cells (Regan et al., 2017), declined (Figures 1C and 1D).

**Figure 2:**
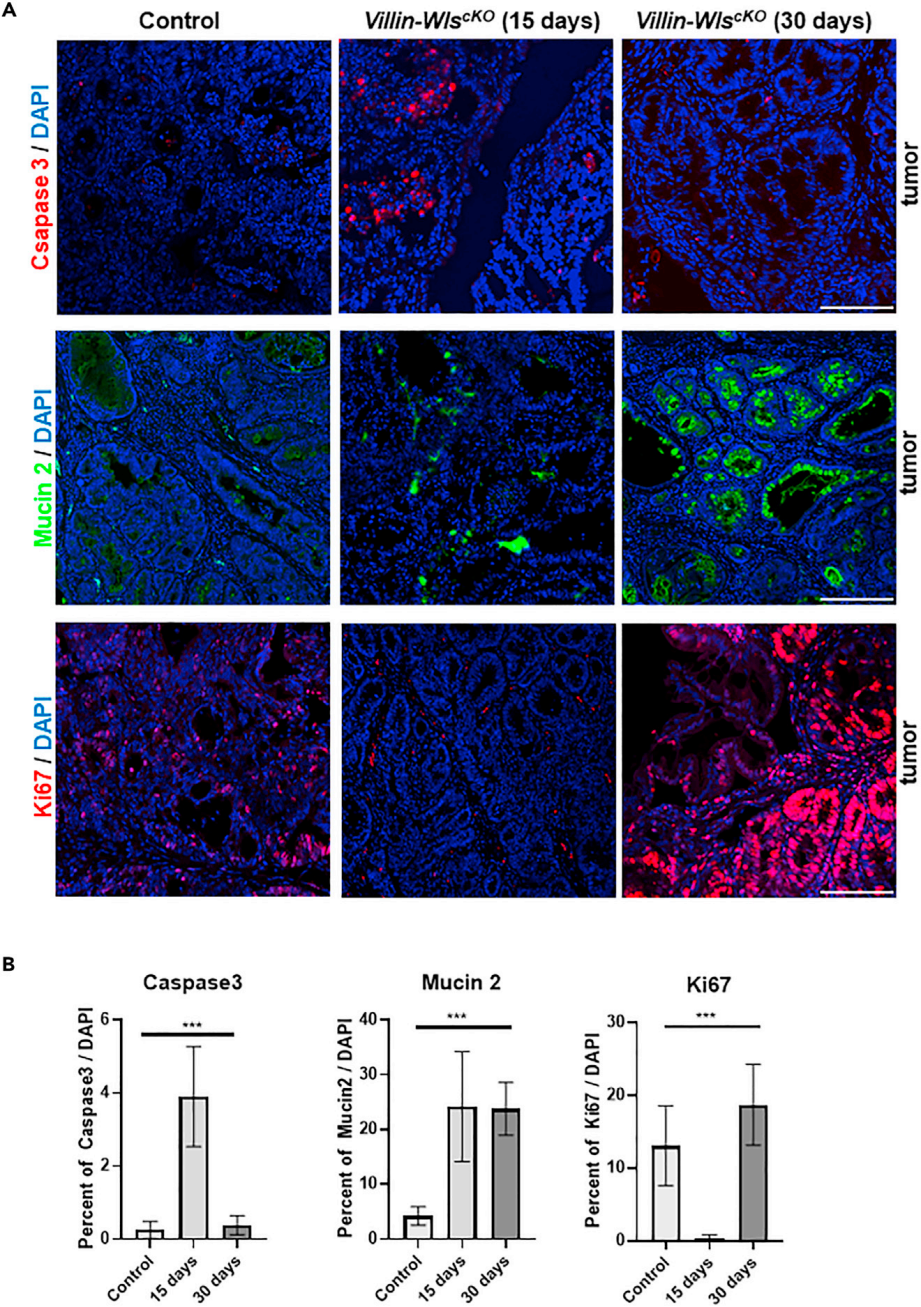
Blocking epithelial Wnt secretion induces apoptosis of AOM/DSS tumor cells and promotes their differentiation (A) Enhanced apoptosis 15 days after blocking the epithelial Wnt secretion (Villin-WlscKO) marked by active/cleaved Caspase3 staining (red, upper panels) is followed by the appearance of more differentiated cells expressing Mucin2 (green, middle panels). In contrast to control AOM/DSS tumor, tumors with 30 days abrogated epithelial Wnt secretion contain an increased number of proliferating cells positive for Ki67 (red, lower panels). Immunohistochemistry (as indicated), DAPI (blue, in all panels) stains nuclei. Scale bars, 100 μm. (B) Quantifications of indicated immunostainings determined as % of positive cells (specific signal to DAPI ratio). One-way ANOVA, graphs show mean ± SD, ∗∗∗p ≤ 0.001.

To acquire further insight, we examined the cellular responses triggered by the loss of epithelial Wnt secretion. Monitoring activated Caspase 3 revealed an extensive tumor cell death 15 days after Wls depletion, whereas at 30 days the number of dying cells returned to level as determined in control with intact epithelial Wnt secretion (Figures 2A, 2B, and S6A). Enhanced apoptosis 15 days after tamoxifen administration was confirmed by TUNEL assay (Figure S6A). Apparently, inactivation of Wnt/β-catenin signaling as a consequence of restricted epithelial Wnt secretion is triggering the elimination of tumor cells. As the size of Villin-Wls^cKO^ tumors did not noticeably differ from control tumors, it is likely that a re-population of the tumor with different cell types occurs. RNA sequencing analysis indicated that *Mucin2 (Muc2)* and *Mucin4 (Muc4)*, markers of terminally differentiated goblet cells, are elevated in Villin-Wls^cKO^ tumors after 30 days. This suggests that tumor cells with abrogated epithelial Wnt secretion are in a more differentiated state. Notably, Muc2-positive goblet cells are present in the Villin-Wls^cKO^ tumors, whereas we rarely saw goblet cells in control tumors (Figures 2A and S6B). Enhanced differentiation marked by Muc2-positive cells followed the apoptotic peak, but in contrast to cellular death, it persisted also after 30 days when it seemed to be most consistent and apparent (Figure 2B). In humans, high expression of MUC2 in colon cancer is attributed to better prognosis and less severe tumors (Betge et al., 2016; Krishn et al., 2016). Moreover, these cells showed extensive proliferation as monitored by Ki67 and PCNA (Figures 2A, 2B, and S6C). The proliferation within Villin-Wls^cKO^ tumors reached its maximum after 30 days, similar to enhanced differentiation. Expression of epithelial Wnts, such as Wnt7a/b, is also strongly reduced or lost suggesting that upon loss of Wnt secretion the repopulation of the tumor occurs via alternate cell types (Figures 1C, 1D, and S6D). Together, we interpret these results to indicate that preventing Wnt secretion in the tumor epithelium triggers apoptosis, which in turn allows the re-population of the tumor tissue by more differentiated cells, such as goblet cells or their precursors. The re-population, preceded by apoptosis of tumor cells, is probably allowed by enhanced proliferation connected with increased differentiation.

**Figure 3:**
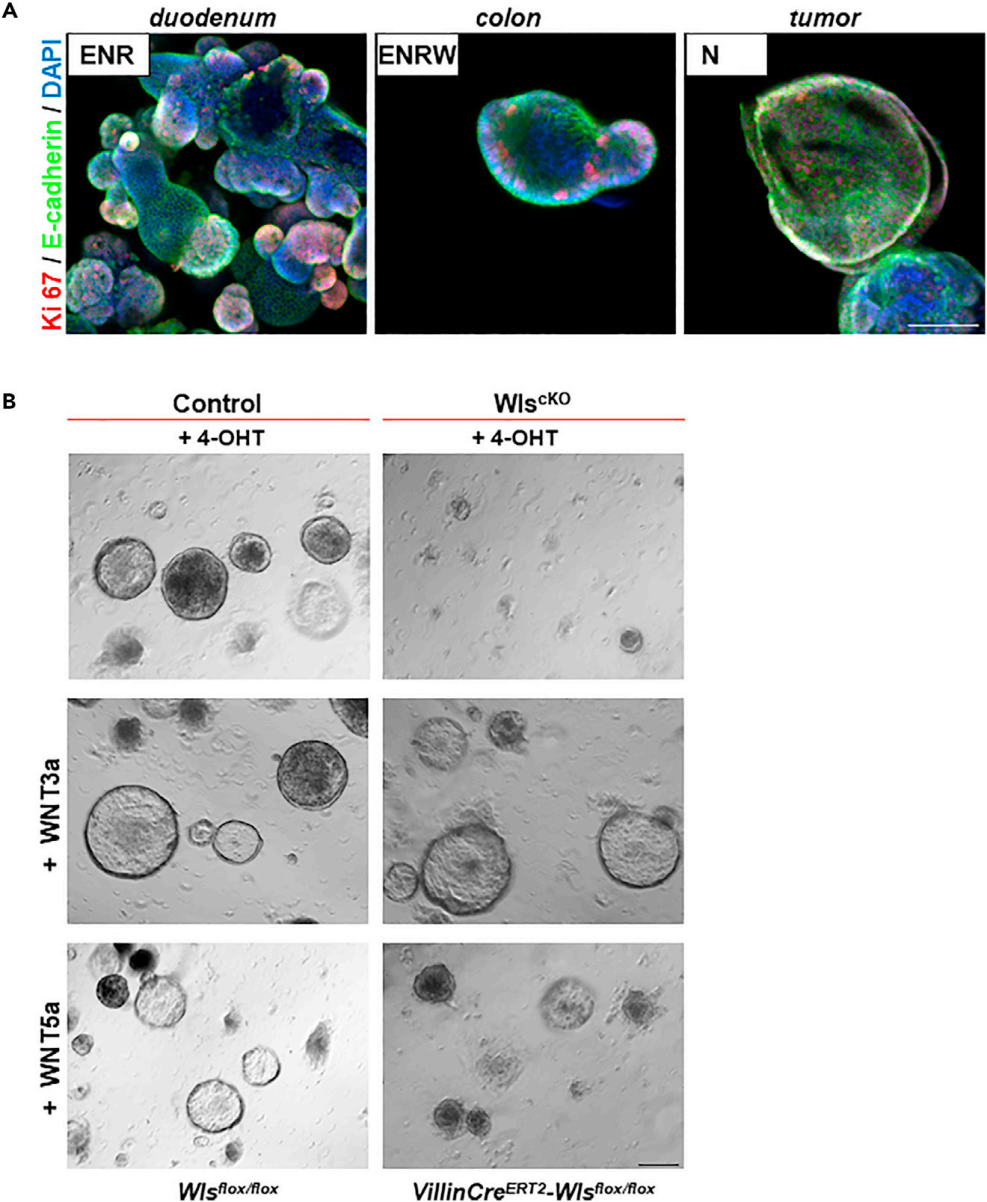
Epithelial Wnts uncouple AOM/DSS tumor cells from mesenchymal stem cell niche and drive/allow tumor growth (A) External activation of Wnt signaling pathway is required for the growth of intestinal organoids. Small intestinal organoids (left panel) depend on addition of RSpondin to the cultivation medium; colonic organoids need external Wnts and RSpondin. In contrast, organoids derived from 3-month-old AOM/DSS tumor cells are able to self-renew in the absence of any external Wnt activators. Organoids were kept in the culture for minimally one month in medium containing supplements as indicated (ENR = EGF, Nogin, RSpondin1; ENRW = ENR + WNT3a, N= Noggin only). Immunohistochemistry showing organoids 2 days after passaging, Ki67 (red) marks proliferating cells; E-cadherin stains cell shapes, and DAPI stains nuclei. (B) AOM/DSS tumor organoids die after the blocking of epithelial Wnt secretion. VillinCreERT2-Wlsflox/flox (WlscKO) or Wlsflox/flox (Control) organoids were treated with 4-OHT to block the expression of Wls and thus Wnt secretion. Addition of 100 ng/mL recombinant WNT3a can rescue the growth of WlscKO organoids. External WNT5a (100 ng/mL) can prolong survival of WlscKO tumor organoids. Shown organoids are 12 days after first administration of 4-OHT (second passage). Scale bar, 50 μm.

The critical role of Wnt expression for the progression of AOM/DSS-induced tumors prompted us to test their role in epithelial organoids derived from these tumors. In a healthy duodenum, Paneth cells provide Wnt ligands for IES cells and therefore organoids from the small intestine do not require exogenous Wnt ligands in their growth medium, whereas colonic organoids can only be maintained by providing external Wnts (Figure 3A). Healthy colonic epithelia do not express any Wnts (Farin et al., 2012). Organoids obtained from AOM/DSS-induced colonic tumors, however, do not require external Wnt ligands because they secrete their own Wnts to promote their growth (Figure 3A). These organoids fully depend on autocrine Wnt secretion, because they die upon addition of 4-OHT, which eliminates *Wls*. Survival and growth were rescued when these *Wls* mutant organoids were supplied with external recombinant Wnt3a ligand (Figure 3B). Hence, endogenously expressed Wnt ligands are responsible for the growth of the colonic AOM/DSS-induced tumors.

## Discussion

Recent studies have put increasing emphasis on the importance of the niche in regulating the behavior of tumor cells, especially regarding the cues that skew the balance between growth and differentiation. First indications that the niche has such a regulatory role were the enhanced expression of Wnt ligands in CRC (Degirmenci et al., 2018a, 2018b; Vider et al., 1996).

The significance of Wnt-producing niche for the progression of colon cancer was also confirmed recently (Mosa et al., 2020). Importantly, inflammation-induced cancer represents a specific subtype of human colon cancer where the alteration of the stem cell niche represents one of the key tumor initiation cues (Guinney et al., 2015; Robles et al., 2016). For example, an increase of Wnt-producing CD34^+^ cells forming the stem cell niche has been proposed to promote inflammation-induced cancer (Koliaraki et al., 2017; Koch, 2017; Stzepourginski et al., 2017). The formation of a cancer stem cell Wnt niche may permit tumor cells to hyperproliferate and to escape from the tight control of the normal stem cell niche. This transient situation may later be fixed by acquiring tumor-autonomous Wnt expression, providing niche independency to the tumor in situations when the inflammation conditions change or disappear.

It has been reported that certain colon cancer cells are still responsive to Wnt ligands despite the presence of pathway-activating mutations in APC or β-catenin (Voloshanenko et al., 2013). Later, distinct types of tumors were discovered, which also exhibit a dependency on Wnt ligands. They harbor mutations in RNF43/ZNRF3 or R-spondins 2/3 translocations (Degirmenci et al., 2018a, 2018b; Giannakis et al., 2014). These mutations occur in a mutually exclusive manner to mutations in APC or β-catenin. Colorectal tumors with mutated RNF43/ZNRF3 or R-spondins 2/3 are sensitive to abrogation of Wnt secretion (Degirmenci et al., 2018a, 2018b; Koo et al., 2012; Madan et al., 2016; Storm et al., 2016).

Here we report the existence of a novel set of Wnt-addicted tumors. In contrast to the cases mentioned earlier, these tumors do not harbor mutations in core components of the Wnt signaling pathway, neither downstream of receptor complex (as *APC*, *Ctnnb1*, *Axin1/2* or *Tcf7l2*/*Tcf4*) nor in the Wnt-enhancing *R-spondin*/*RNF43*/*ZNRF3* branch. Importantly, these tumors fully depend on hyperactivated Wnt signaling triggered by autosecreted Wnt ligands. A connection of tumors with colon inflammation is supported by the fact that the mutational landscape of inflammation-induced colon cancer differs from the majority of sporadic colon tumors, which typically harbor mutations in *APC* or *Ctnnb1* (Guinney et al., 2015; Network CGA, 2012; Robles et al., 2016; Sanchez-Vega et al., 2018). The dependency on hyperactivated Wnt signaling seems to be the characteristic feature of various epithelial tumors independent of the cause of this hyperactivation (Steinhart et al., 2017; Tammela et al., 2017; Zimmerli et al., 2018). One way to achieve Wnt hyperactivation is aberrant Wnt secretion, as described here. On the other hand, the key role of acquired epithelial Wnt secretion in human inflammation-induced CRC should be further validated.

Intriguingly, also in humans there are CRC cases that do not harbor obvious mutations in Wnt transduction components. We analyzed data from such patients that lack mutations in the following core components: APC, CTNNB1, RNF43, ZNRF3, TCF7L2 (TCF4), FZD1, FZD3, DKK3, and LRP6. These CRCs exhibit robust expression of various Wnt ligands (Figures 1A and 1B), raising the possibility that our findings in the mouse have a clinical counterpart in humans, independent of the way how CRC was initiated.

In summary, we learned that Wnt ligands are involved in governing the initiation and growth of colonic tumors that do not harbor common mutations in components of the Wnt/β-catenin signaling pathway. When Wnt ligand secretion is prevented in such tumors, Lef1 cells start to die and are eliminated from the tumor. Interestingly, the tumor is then re-populated by more differentiated cells, raising the possibility that blocking Wnt secretion may be a promising therapy in such type of colon tumors. Despite clear evidence pointing to the activity of acquired epithelial Wnts in progression of inflammation-induced colon cancer model, the mechanism triggering epithelial Wnt secretion remains unknown and was not a major focus of this study. The broad spectrum of possibilities responsible for this phenomenon from mutational activation of some signaling pathways or transcription factors to epigenetic changes in Wnt regulatory regions may be a focus of subsequent studies.

### Limitations of the study

Murine colon cancer experiments were performed on C57BL/6 background. As mentioned in the result section, tumors and tumor organoids based on other genetic background may harbor different mutations, including cancer-driving mutations. Despite the clear impact of acquired epithelial Wnt secretion on the progression of AOM/DSS tumors the mechanism of how epithelial Wnt secretion is achieved needs to be determined.

The analysis of human CRC data just suggests the existence of colon cancer types lacking the mutations in core Wnt signaling components that depend on Wnt ligands. However, the analysis does not allow to distinguish epithelial and non-epithelial Wnts and the type/class of colon cancer (e.g., if tumors were inflammation induced).

## STAR★Methods

### Key resources table

**Table:** 

REAGENT or RESOURCE	SOURCE	IDENTIFIER
**Antibodies**		

E-cadherin	BD Transduction Laboratories	Mouse monoclonal, Cat#610182 RRID: AB_397581
Ki67	Abcam	Rabbit polyclonal, Cat# ab15580, RRID:AB_443209
Muc2 (Mucin2)	Santa Cruz	Rabbit polyclonal, H-300, Cat# sc-15334, RRID:AB_2146667
Wnt7b	Novus biologicals	Rabbit polyclonal, Cat# NBP1-59564, RRID:AB_11005472
Cleaved caspase 3 (Asp175)	Cell Signaling	Rabbit, Cat#9661S, RRID:AB_2341188
Lef1	Cell Signaling	Rabbit monoclonal (C12A5), Cat#2230S, RRID:AB_823558
PCNA	Abcam	Rabbit polyclonal, Cat# ab18197, RRID:AB_444313
Alexa Fluor 594 goat anti-rabbit	Thermofisher	Cat # A-11012
Alexa Fluor 647 goat anti-mouse	Thermofisher	Cat# A-21235

**Chemicals, peptides, and recombinant proteins**		

Gentle cell dissociation reagent	Stemcell Technologies	07174
Advanced DMEM/F12 (1X)	Gibco	12634–010
Collagenase D	Sigma (Roche)	11088866001
Dispase	Gibco	17105-041
Heat Inactivated FBS	Gibco	10500-064
TrypLE Express	Gibco	12604-013
Tween 20	Sigma	P1379-250ML
Bovine Serum Albumin raction V	Sigma	10735086001
Paraformaldehyde solution 4% in PBS	ChemCruz	sc-281692
FluorSave reagent	Millipore	345789-20ML
PBS, pH 7.4 (1X)	Gibco	10010-015
DAPI Solution (1 mg/mL)	ThermoFisher	62248
Matrigel for organoid culture	Corning	FAL356255
Glutamax	ThermoFischer	35050-061
HEPES (1M)	ThermoFischer	15630-056
Pen/strep	ThermoFischer	15140-122
N-acetylcystein	Sigma	A9165-5g
B27 (50×)	ThermoFischer	17504-044
N2 (100×)	ThermoFischer	17502-048
EGF (mouse, recombinant)	ThermoFischer	PMG8041
Noggin (mouse, recombinat)	Sigma	SRP3227-20ug
mRspondin1 (murine, recombinant)	Peprotech	315-32
L-Wnt3a cells	ATCC	CRL-2647

**Critical commercial assays**		

*In Situ* cell death detection kit, TMR red	Sigma	Cat#12156792910

**Deposited data**		

Exome sequencing	This paper	ENA: PRJEB45932 (http://www.ebi.ac.uk/ena/data/view/PRJEB45932)
Bulk RNAseq data	This paper	GEO: GSE181738

**Experimental models: Organisms/strains**		

Mouse: Wls-flox (Wls><GM><B6-Wls(E1flox)<tm1Basler)	Valenta et al., 2016	NA
Mouse: Villin-CreERT2 (B6.SJL-Tg(Vil-cre/ESR1) 23Syr)	El Marjou et al., 2004	RRID:MGI: 3053826

**Software and algorithms**		

FastQC	Babraham Bioinformatics	http://www.bioinformatics.babraham.ac.uk/projects/fastqc
TopHat v2.1.1	Trapnell et al. (2009)	https://ccb.jhu.edu/software/tophat/index.shtml
Cufflinks (v2.2.1)	Trapnell et al., 2010	http://cole-trapnell-lab.github.io/cufflinks/
ggplot2 (v2.2.1)	Wickham, 2016	https://cloud.r-project.org/web/packages/ggplot2/index.html
Ggrepel	CRAN.R-project	https://CRAN.R-project.org/package=ggrepel
ImageJ (FIJI) v. 2.0.0	Schindelin et al., 2012	https://imagej.net/Fiji/
R Software 3.6.1	GNU project	https://www.r-project.org
R studio	RStudio	https://www.rstudio.com
GraphPad prism v7.0a	GraphPad Software Schneider	https://www.graphpad.com/scientific- software/prism/

### Resource availability

#### Lead contact

Further information and requests for resources and reagents should be directed to and will be fulfilled by the lead contact, Konrad Basler (konrad.basler@imls.uzh.ch).

#### Materials availability

This study did not generate new unique reagents.

### Experimental model and subject details

#### Mouse experiments

Mouse experiments were performed in accordance with Swiss guidelines and approved by the Veterinarian Office of Kanton Zürich, Switzerland. AOM/DSS tumors were induced as was previously described (Moor et al., 2015). Briefly, Azoxymethane (AOM) was injected intra-peritoneally (12.5 mg/kg), after 7 days animals were treated for five days dextran sulfate sodium (DSS, 2% in drinking water) followed by two weeks recovery time. DSS treatment with recovery period was repeated additionally two times (in total 3× DSS treatment, Figure 1C). Tumors were collected 120 days after AOM injection. To achieve the conditional deletion of Wls (Wls^cKO^) the *Wls* conditional allele (Valenta et al., 2016) was combined with *Villin-Cre*^*ERT2*^ driver. For Cre-mediated recombination tamoxifen (Sigma) was injected (80 mg/kg) intraperitoneally for 5 consecutive days starting the first injection 15 or 30 days before tumor collections. Preferentially females were used aged between 6 to 10 weeks at the beginning of the experiment. All animals were on C57Bl6 background.

#### Intestinal organoids

Intestinal duodenal and colonic organoids were generated and cultured as described (Degirmenci et al., 2018a, 2018b; Sato and Clevers, 2013; Valenta et al., 2016). Organoids were cultivated in medium supplemented as follows: duodenal organoids in ENR = EGF (Gibco/Thermofisher) 50 ng/mL, Noggin (Sigma) 100 ng/mL, R-spondin1(Sigma) 500 ng/mL; colonic organoids in ENRW = ENR + 30% Wnt3a conditioned medium, tumor organoids N= Noggin (Sigma), 100 ng/mL. Organoids from AOM/DSS tumors were generated from 3 months old AOM/DSS tumors as described by Xue and Shah (2013) and cultured for first week full medium for colon organoids (ENRW), followed by cultivation in medium lacking Wnt3a, R-spondin1 and EGF. The tumor organoids can grow in such a medium for minimally 4 weeks. Tumor organoids were generated from tumors of *Wls*^*flox/flox*^ and *Villin-Cre*^*ERT2*^*,Wls*^*flox/flox*^ animals. To achieve Cre-mediated recombination and thus Wls elimination control (*Wls*^*flox/flox*^) and Villin-Wls^cKO^ (*Villin-Cre*^*ERT2*^*,Wls*^*flox/flox*^) organoids were treated with (Z)-4-Hydroxitamoxifen (4-OHT, Sigma), 500 ng/mL 24 hour after passaging for 12 hours then let to grow till splitting, passaged and treated again with 4-OHT for 12 hours.

### Method details

#### Histology and immunohistochemistry and immunocytochemistry

Freshly isolated tumors were fixed in 4% paraformaldehyde, dehydrated and mounted in paraffin using standard protocols. Standard immunohistochemical protocols were performed with the following antibodies (diluted 1:100): mouse anti-E-cadherin (BD Transduction Laboratories), rabbit anti-Ki67 (Abcam), Wnt7b (recognizing both Wnt7a and Wnt7b) (Novus Biological), Cleaved Caspase 3 (Cell Signaling), LEF1 (Cell Signaling), MUCIN2 (Santa Cruz). TUNEL staining was performed using In Situ Cell Death Detection Kit,TMR red (Roche, distributed by Sigma).

Secondary antibodies (dilution1:400) were anti-rabbit, anti-mouse, anti-chicken, antibodies conjugated with Alexa (A488, A594 or A647) from ThermoFisher Scientific. Images were taken using a Leica LSM 710 or Leica SP8 confocal microscope and processed using ImageJ (FIJI) and AdobePhotoshopCS6 software. Unless it is otherwise stated, tumor from minimally 6 independent animals (2-3 tumor per each animal) were checked and representative pictures are shown.

### Quantification and statistical analysis

Signal quantification was done as follows: Fiji software (version 2.1.0/1.53c) was used to quantify the amount of fluorescent signal on the original Z-stack images. First, the individual channels manual threshold using Huang thresholding method was set up. Subsequently, volumes of signals were counted for each channel. Antibody staining signal was related to DAPI reference signal by dividing. One-way ANOVA was used for statistical analysis.

#### RNA isolation, RNAsequencing, DNA exome sequencing and analysis

RNA from tumors was isolated and processed as described (Moor et al., 2015; Valenta et al., 2016). RNA sequencing (RNAseq) including library preparation was done performed by by Genomics Platform, University of Geneva, Geneva, Switzerland. The quality of the reads was checked using FastQC (http://www.bioinformatics.babraham.ac.uk/projects/fastqc). For the analysis of the reads, Tuxedo protocol was used with default parameters (Trapnell et al., 2012). The reads were mapped against NCBI *Mus musculus* reference genome (GRCm38.p5) using TopHat v2.1.1 (Trapnell et al., 2009). The output files obtained from TopHat were given to Cufflinks (v2.2.1) as inputs to assemble transcripts and quantify their expressions (Trapnell et al., 2010). After merging the transcripts with Cuffmerge, Cuffdiff was used to detect differentially expressed genes between two conditions (Wild Type vs. Villin-Wls^cKO^). At the same time, Cuffdiff was used again without merging the transcripts to enable sample-based comparisons.

From the Cuffdiff results, FPKM (fragments per kilobase of transcript per million mapped reads) values for the selected genes of interest were filtered to create a heatmap using pheatmap (v1.0.8) of R (http://CRAN.R-project.org/package=pheatmap, https://www.R-project.org). For hierarchical clustering of these selected genes, pearson correlation was performed. Moreover, in order to show whether the selected genes are downregulated or upregulated, average FPKM value for Villin-Wls^cKO^ samples was compared to wild type average value. The result is shown as a bar next to the heatmap.

A volcano plot is generated with the help of ggplot2 (v2.2.1) (http://ggplot2.org) and ggrepel (https://CRAN.R-project.org/package=ggrepel) in which the selected genes are shown with labels. Log2 (Fold Change) and -log10(q-value) of the genes were plotted. The data points were labelled significant if their q-value is smaller than 0.05. The minimum q-value that TopHat can calculate is fixed. Hence, there is a cluster of genes in the upper side of volcano plot where the minimum q-value is reached for the most significantly differentially expressed genes.

RNA for cDNA of *Wnt* genes was isolated and reversely transcribed as described (Valenta et al., 2016). Primers used for PCR with GoTaq G2 DNA polymerase (Promega) were as in Farin et al. (Farin et al., 2012).

DNA from three different tumors and two tumor organoids was isolated using DNA Easy Blood&Tissue kit (Quiagen), exome sequencing including library preparation was performed by Genomics Platform, University of Geneva, Geneva, Switzerland.

The sequence reads of all 5 samples were aligned to the reference genome of *Mus musculus* (mm10) using BWAmem (v.0.7.15), after which the alignments were cleaned using the GATK (v3.7.0) and PICARD (v 2.9.0) software packages. For the alignment cleaning, first indels were realigned using RealignerTargetCreator and IndelRealigner, followed by fixing mate information using FixMateInformation and subsequently library and optical duplicates were removed using MarkDuplicates and all base scores were recalibrated using BaseRecalibrator and PrintReads. Next, single nucleotide variants and indels were called on the cleaned alignments of all 5 samples using SAMTOOLS's mpileup application (v.1.4). Variants having a QUAL and GQ smaller than 30 and 99, respectively, coverage <20×, mutation allele frequency of <10% and dbSNP record were removed from the variant list. Additionally, we removed all variants that did not appear in all 5 samples. The remaining variants with snpEff annotations can be found as a table (Data S1).

#### Analysis of human CRC datasets

To compare our results with the human colon cancer data, Colon Adenocarcinoma (Weinstein et al., 2013) dataset was selected from cBioPortal database (Cerami et al., 2012; Gao et al., 2013). Tumor samples that do not have a mutation in a given list of genes (APC, CTNNB1, RNF43, ZNRF3, TCF4, FZD1, FZD3, DKK3, LRP6) were compared with samples having at least one mutation in these genes. To do so, FPKM expression files for each sample were downloaded from The Cancer Genome Atlas (TCGA) (Weinstein et al., 2013; Grossman et al., 2016). In the dataset obtained from cBioPortal, 93% of the samples have at least one mutation in the genes mentioned above. In the remaining 7% of the samples, the expression of the significant genes from the Wilcoxon rank-sum test were examined. FPKM values higher than 0.1 were accepted as an indication of expression. The expression distribution of the Wnt genes are shown in the pie chart using Plotrix (v3.7-2) package from R.

## Data Availability

• RNA-seq data have been deposited at GEO. Exome sequencing data have been deposited at ENA (European Nucleotide Archive). Accession numbers for both RNA-seq and Exome sequencing datasets are listed in the Key resources table. Microscopy and other data reported in this paper will be shared by the lead contact upon request. • No unique code was created within this study. • Source data underling Figures S2 and S3 containing the list of common mutations within coding regions determined by exome sequencing in 3 different AOM-DSS tumors and 2 epithelial organoids derived from AOM-DSS tumors is provided in Data S1. Source data underling Figures 1C and 1D showing the list of genes downregulated (down) or upregulated (up) upon blocking epithelial Wnt secretion in AOM-DSS tumors (30 days after tamoxifen injection) are indicated in Data S2. For analysis 5 villin-Wls^cKO^ tumors were compared to 5 control ones, summary results are shown. q-value 0.0005. Any additional information required to reanalyze the data reported in this paper is available from the lead contact upon request.

## Supplemental information

Document S1. Figure S1–S6

Data S1. The list of common mutations determined in AOM/DSS tumors, related to Figures S2 and S3 The table contains the list of common mutations within coding regions determined by exome sequencing in 3 different AOM/DSS tumors and 2 epithelial organoids derived from AOM-DSS tumors

Data S2. The list of upregulated and downregulated genes upon blocking Wnt secretion in AOM/DSS tumors, related to Figures 1C and 1D The table shows the list of genes downregulated (down) or upregulated (up) upon blocking epithelial Wnt secretion in AOM-DSS tumors (30 days after tamoxifen injection). For analysis 5 villin-Wls^cKO^ tumors were compared with 5 control ones; summary results are shown. q value, 0.0005. Any additional information required to reanalyze the data reported in this article are available from the lead contact upon request
