# Supplementary material for: Epithelial Wnt secretion drives the progression of inflammation-induced colon carcinoma in murine model
Source: iScience. 2021 Oct 28;24(12):103369. doi: 10.1016/j.isci.2021.103369 (PMC8607204; doi:10.1016/j.isci.2021.103369)
Supplement: Document S1. Figure S1–S6 [file mmc1.pdf]

iScience, Volume *24*

Supplemental information

Epithelial Wnt secretion drives the progression of inflammation-induced colon carcinoma in murine model

Bahar Degirmenci, Cansu Dincer, Habibe Cansu Demirel, Linda Berkova, Andreas E. Moor, Abdullah Kahraman, George Hausmann, Michel Aguet, Nurcan Tuncbag, Tomas Valenta, and Konrad Basler

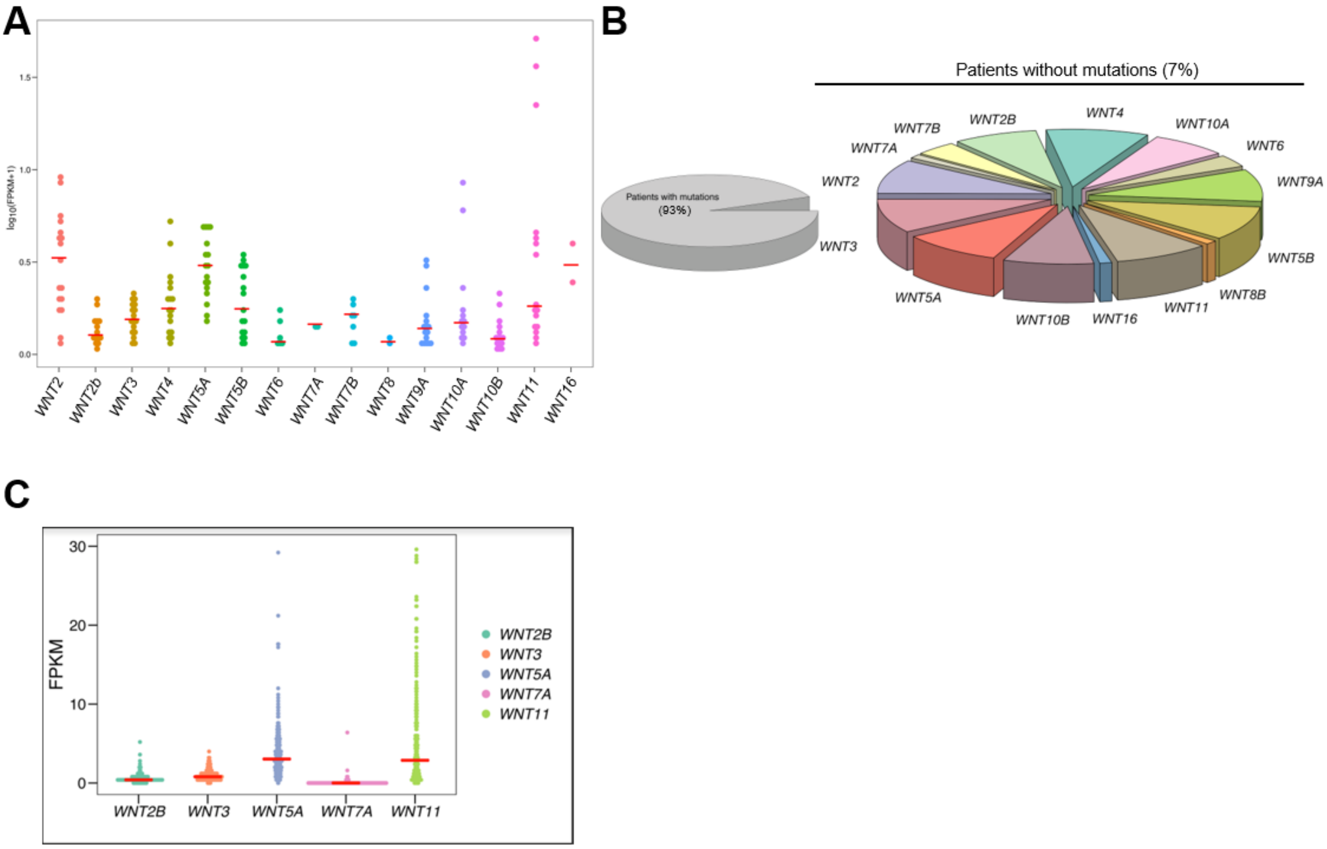

Figure S1 (related to Figure 1)

The expression of Wnt ligands in human colon tumors lacking mutations in core Wnt signaling components.

A)Human tumors devoid of mutations in core Wnt signaling components express various Wnt ligands. Graph shows the expression levels of indicated Wnts as $\log_{10}$FPKM+1 (fragments per kilobase million+1). Each dot represents one patient case, TCGA(PanCancer Atlas) was used as a source of data. B) Wnt ligands are expressed differently by colon tumors lacking the mutation in core Wnt signaling (7% of all colon cancer cases; 93% harbors mutation in core Wnt sinaling components). C) Human tumors harboring mutations in core Wnt signaling components express various Wnt ligands. Graph shows the expression levels of indicated Wnts as FPKM (fragments per kilobase million). Each dot represents one patient case, TCGA(PanCancer Atlas) was used as a source of data.

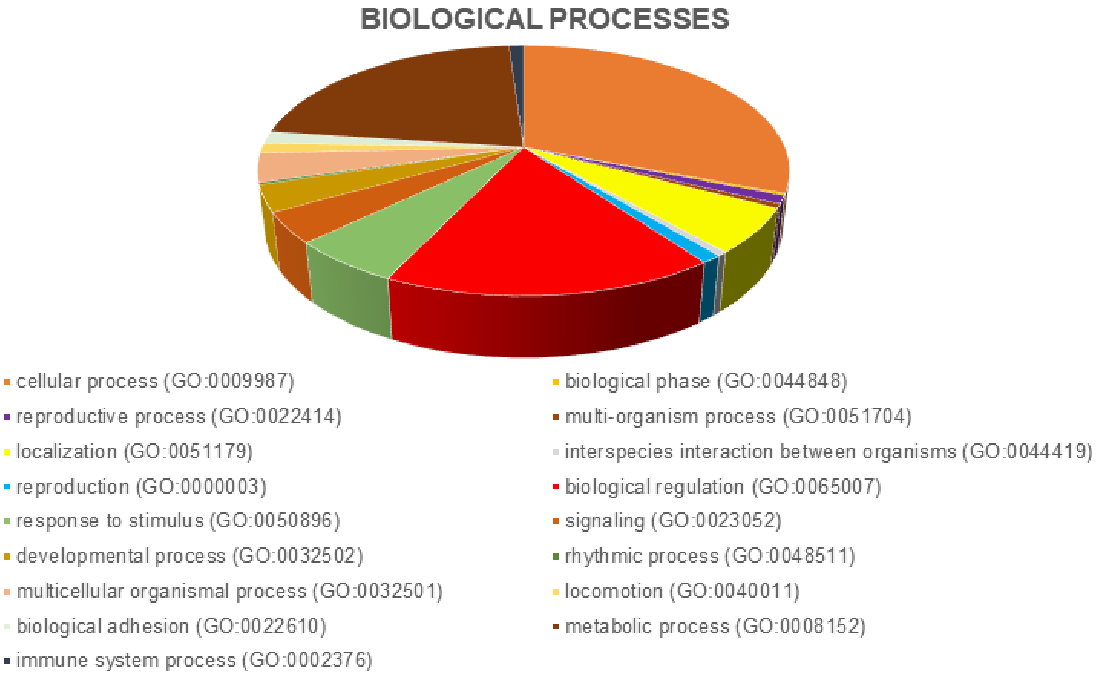

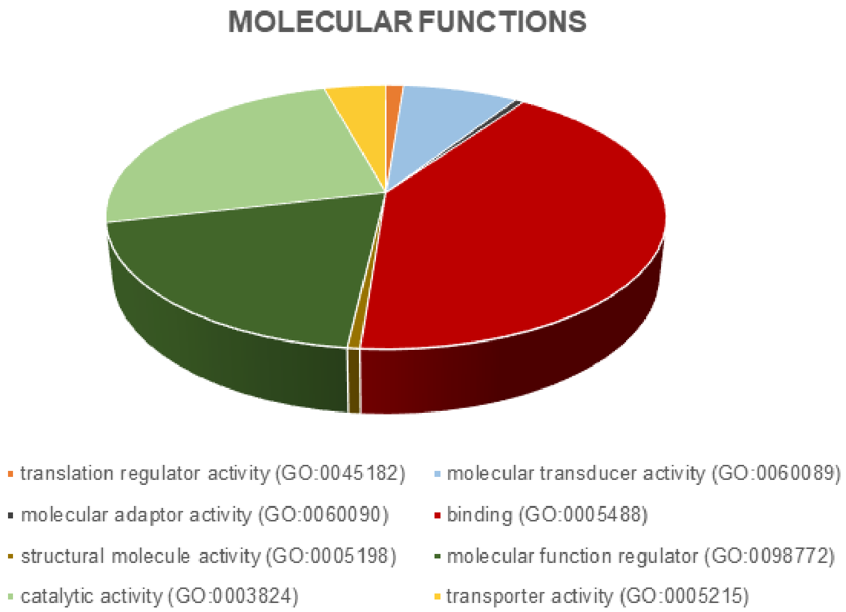

Figure S2 (related to Figure 1)
Complex mutational landscape in AOM/DSS tumors.
Non-synonymous mutations found in AOM/DSS tumors affect the coding regions of genes involved in various biological processes and having distinct molecular function. In total, 199 genes were found to harbor at least one non-synonymous mutation (in all three analyzed tumors and two tumor-derived organoids) in comparison to reference genome of C57Bl6 mouse strain. PANTHER gene ontology (GO) classification system was used to probe genes that showed mutations in coding regions determined by exome sequencing.

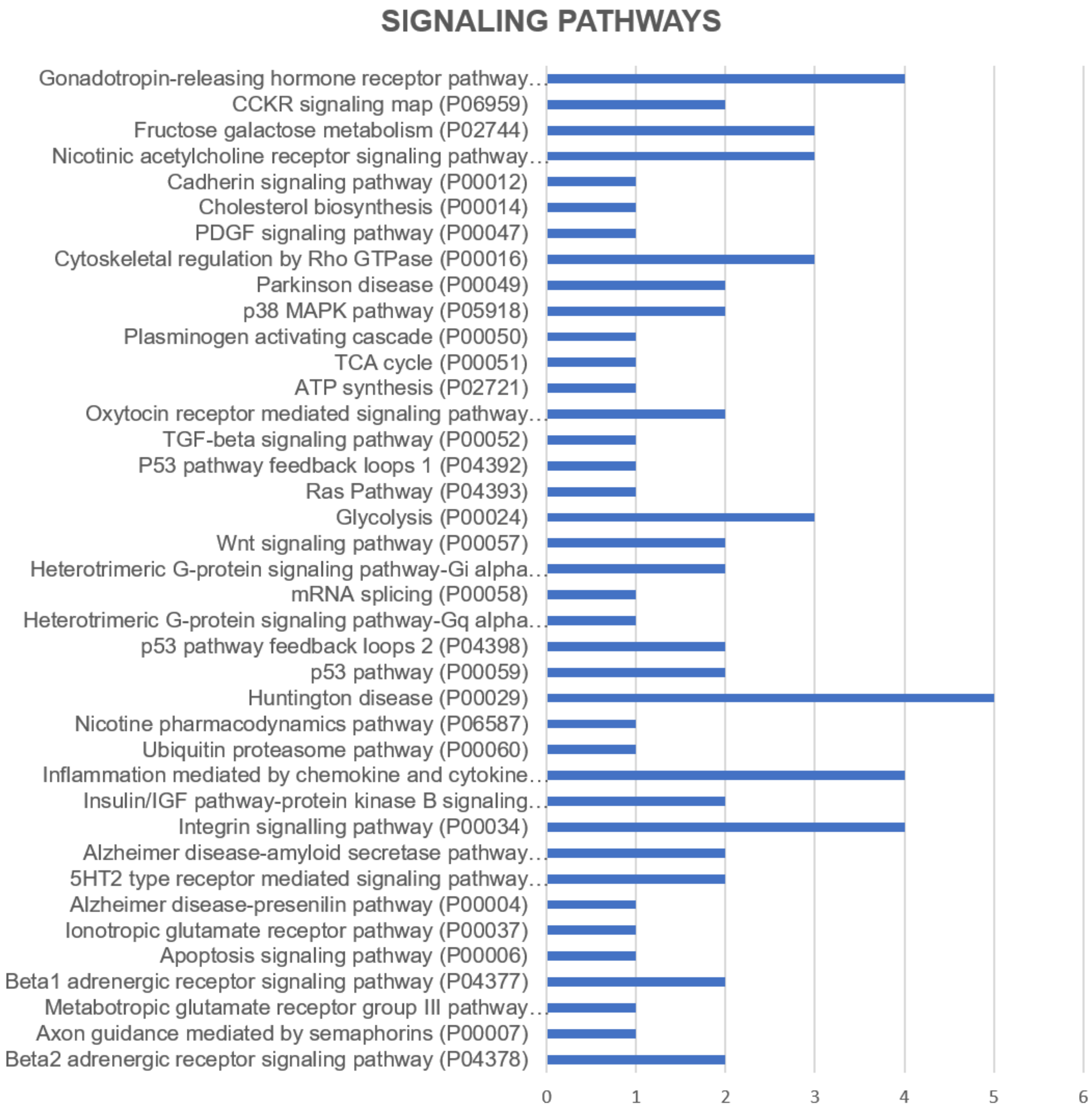

Figure S3(related to Figure 1)
Complex mutational landscape in AOM/DSS tumors – signaling pathways.
Non-synonymous mutations found in AOM/DSS tumors affect the coding exons of genes playing role within various signaling pathways. As in previous figure, PANTHER gene ontology (GO) classification system was used to probe genes that showed mutations in coding regions determined by exome sequencing. From 199 mutated genes to 73 of them a role in signaling pathway can be attributed. In the case of Wnt pathway no mutation in core components was determined. Two genes indicated as connected to Wnt signaling were *Siah1* and *Senp2*, both non-essential components.

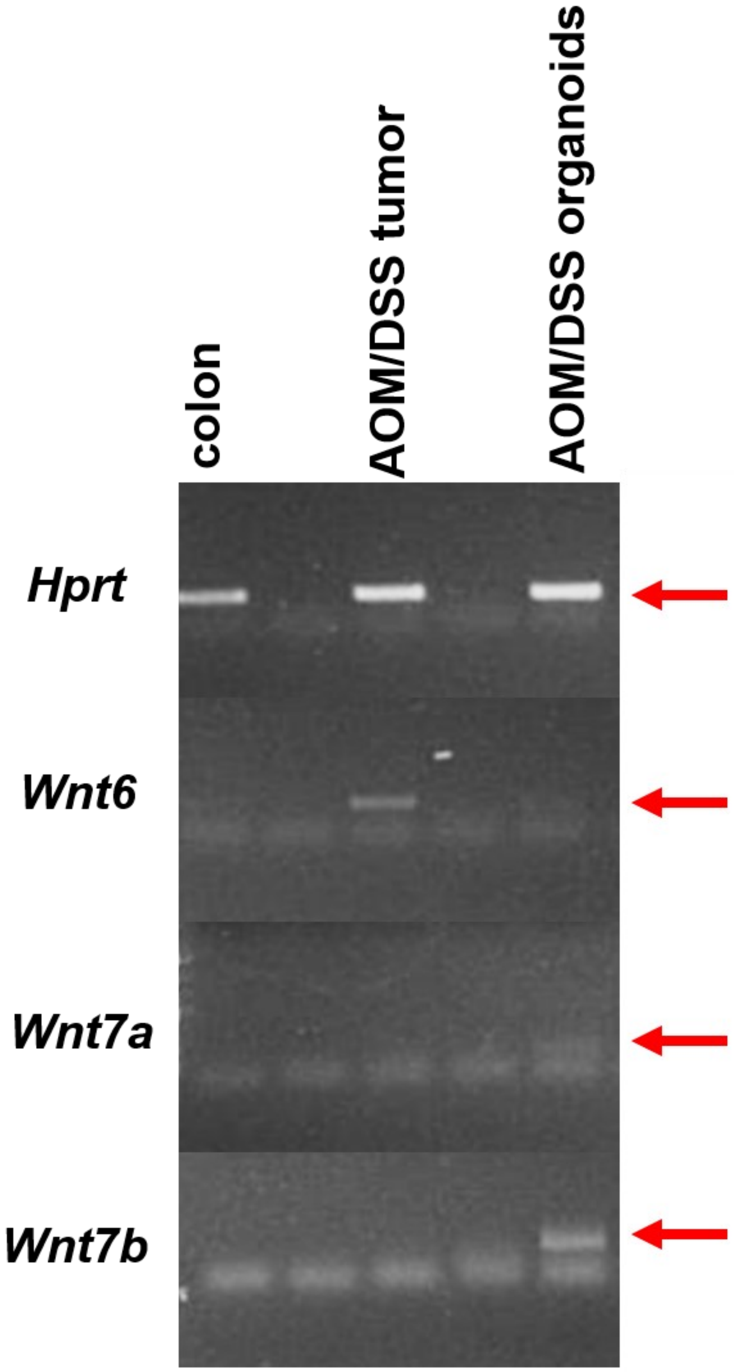

Figure S4 (related to Figure 1)
Wnt7a, Wnt7b are produced by tumor epithelium.
Expression of *Wnt* genes in the colon, AOM/DSS tumors and epithelial organoids derived from AOM/DSS tumor. Reverse-transcription PCR using cDNA from isolated as indicated. cDNA pools were normalized to *Hprt* expression levels; PCR cycle number was 30. Arrows indicated the size/expected position of particular PCR product.

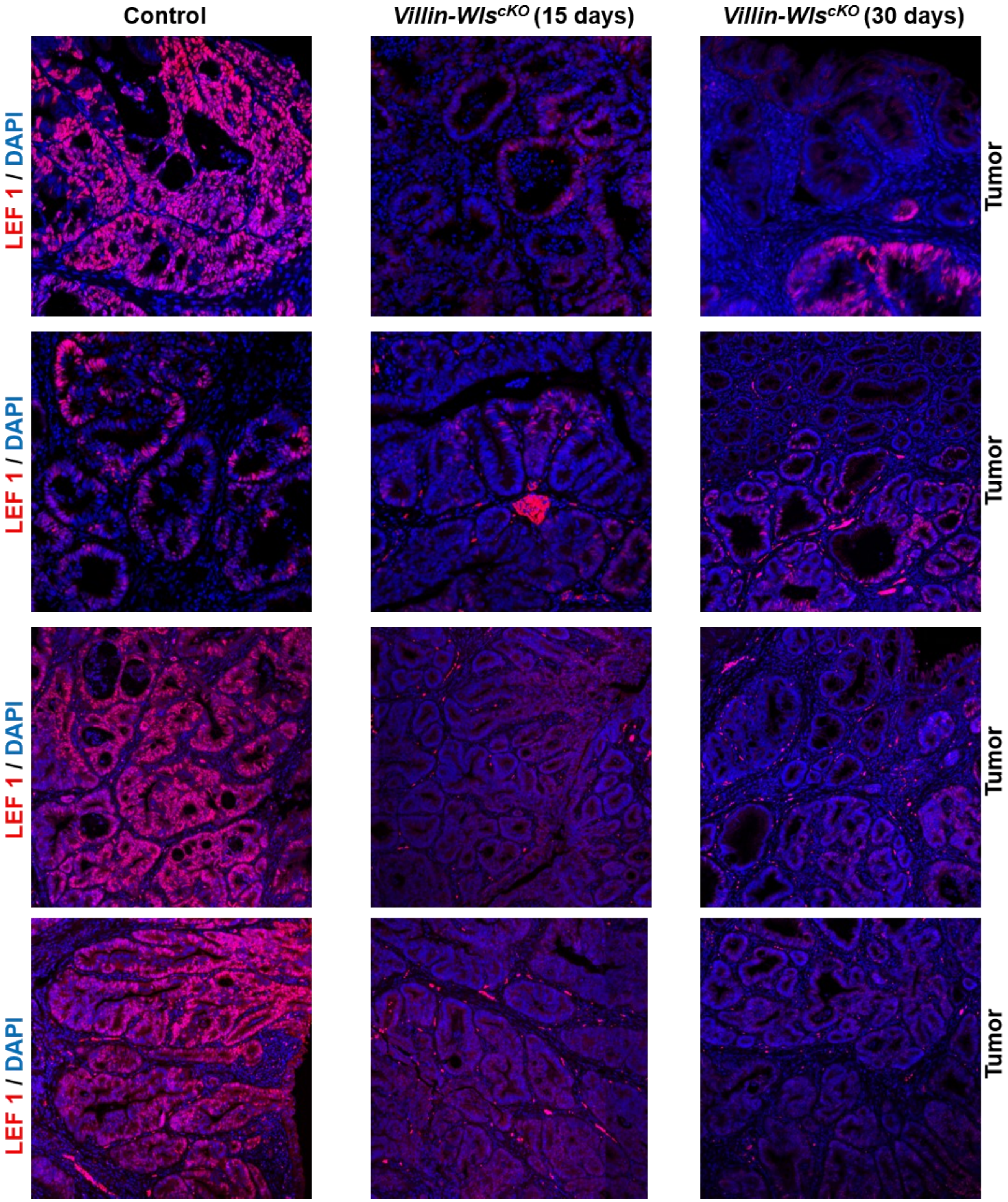

Figure S5 (related to Figure 1)
Reduced expression of colon cancer marker LEF1 in multiple tumors after preventing Wnt-secretion from tumor epithelium.
Immunohistochemistry: LEF1(red), DAPI (blue) indicates nuclei. Time after first Tamoxifen injection is indicated. Numbers indicate different individual tumors.

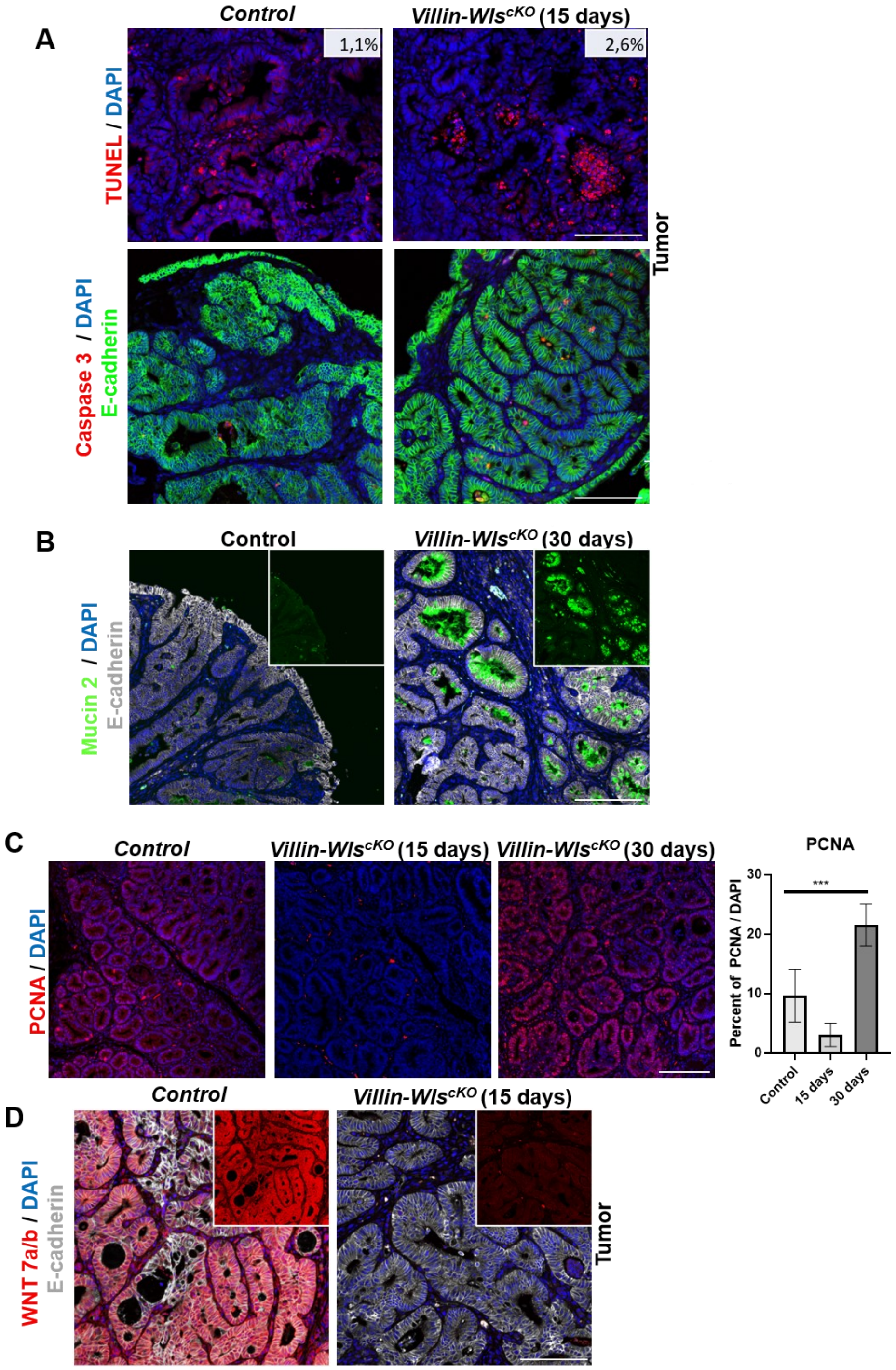

Figure S6 (related to Figure 2)

Enhanced tumor differentiation is connected to reduced expression of WNT7a/b after preventing Wnt-secretion from tumor epithelium.

A) Enhanced apoptosis in AOM-DSS tumors upon blocking epithelial Wnt-secretion 15 days after the tamoxifen administration. TUNEL (red, upper panel) or cleaved-Caspase3 (red, lower panel) indicate apoptotic cells, E-cadherin (green, lower panel) marks epithelial (including tumor) cells. The numbers in upper panels show % of positive cells (specific signal to DAPI ratio). B) Enhanced apoptosis is followed by promoted differentiation determined by appearance of Mucin2 (green) positive cells. Insets show single green channels (Mucin2). E-cadherin (gray) denotes epithelial cells. C) Increased proliferation marked by PCNA (red) 30 days after blocking Wnt-secretion. Quantifications of indicated immunostainings determined as % of positive cells (specific signal to DAPI ratio). One-way ANOVA, graph shows mean ± SD, *** p-value ≤ 0.001. (n=4 tumors for each parallel respectively each time point in the case of Villin-Wls^{cKO}). D) Blocking epithelial Wnt-secretion results in reduced expression of Wnt7a/b in AOM-DSS tumors 15 days after tamoxifen injection. Immunohistochemistry for WNT7a/b (red), E-cadherin (gray) marks epithelial cells. Insets show WNT7a/b staining (red).

For all panels: DAPI (blue) stains nuclei. Scale bar = 100 μm.
